# Veränderung der psychischen Belastung in der COVID-19-Pandemie in Deutschland: Ängste, individuelles Verhalten und die Relevanz von Information sowie Vertrauen in Behörden

**DOI:** 10.1007/s00103-021-03278-0

**Published:** 2021-01-22

**Authors:** Eva-Maria Skoda, Anke Spura, Freia De Bock, Adam Schweda, Nora Dörrie, Madeleine Fink, Venja Musche, Benjamin Weismüller, Anke Benecke, Hannah Kohler, Florian Junne, Johanna Graf, Alexander Bäuerle, Martin Teufel

**Affiliations:** 1 Klinik für Psychosomatische Medizin und Psychotherapie, LVR-Klinikum Essen, Universität Duisburg-Essen, Virchowstr. 174, 45147 Essen, Deutschland; 2 Bundeszentrale für gesundheitliche Aufklärung (BZgA), Köln, Deutschland; 3 Abteilung für Psychosomatische Medizin und Psychotherapie, Medizinische Universitätsklinik, Universität Tübingen, Tübingen, Deutschland

**Keywords:** SARS-CoV‑2, Generalisierte Angst, Depression, Psychischer Distress, Pandemiephasen, SARS-CoV‑2, Generalized anxiety, Depression, Psychological distress, Pandemic phases

## Abstract

**Hintergrund:**

Auswirkungen der COVID-19-Pandemie auf die psychische Gesundheit zeigten sich bereits früh. Das Ausmaß der Auswirkungen, insbesondere kumulativ über die lang anhaltende Zeit der Pandemie, ist für Deutschland noch nicht umfassend untersucht worden.

**Ziel der Arbeit:**

Ziel der Studie war es, psychische Belastungen sowie COVID-19-bezogene Erlebens- und Verhaltensweisen zu erheben und deren Veränderung über die verschiedenen Phasen der Pandemie in Deutschland darzustellen.

**Material und Methoden:**

In die deutschlandweite onlinebasierte Querschnittsstudie (10.03.–27.07.2020) konnten 22.961 Menschen eingeschlossen werden (Convenience Sample). Erhoben wurden: generalisierte Angst (GAD-7), Depression (PHQ-2), psychischer Distress (DT) sowie COVID-19-bezogene Erlebens- und Verhaltensweisen wie *COVID-19-bezogene Angst, Vertrauen in staatliche Maßnahmen, subjektives Informiertheitslevel, adhärentes Sicherheitsverhalten *und* persönliche Risikoeinschätzung für Ansteckung/Erkrankungsschwere*. Die Pandemie wurde retrospektiv in 5 Phasen (Anfangs‑, Krisen‑, Lockdown‑, Neuorientierungsphase und „neue Normalität“) eingeteilt.

**Ergebnisse:**

Es zeigten sich im Vergleich zu Prä-COVID-19-Referenzwerten anhaltend erhöhte Werte in GAD‑7, PHQ‑2 und DT. *COVID-19-bezogene Angst, Informiertheitslevel, Vertrauen, Sicherheitsverhalten *und* die Einschätzung, an COVID-19 zu erkranken,* zeigten, nach initial starkem Anstieg, einen Abfall bis z. T. unter den Ausgangswert. Ausnahme waren konstante *Einschätzungen, einen schweren Verlauf von COVID-19 zu haben* bzw. *daran zu versterben*.

**Diskussion:**

Die durch alle Pandemiephasen anhaltend erhöhten Werte psychischer Belastung verdeutlichen die Notwendigkeit nachhaltiger Unterstützungsangebote. Sinkende Werte in Bezug auf Vertrauen in staatliche Maßnahmen und das subjektive Informiertheitslevel unterstreichen das Gebot gezielter Aufklärung.

**Zusatzmaterial online:**

Zusätzliche Informationen sind in der Online-Version dieses Artikels (10.1007/s00103-021-03278-0) enthalten.

## Abstract (en)

**Background:**

The potential impact of the COVID-19 pandemic on mental health was evident early on. The extent of the effects, especially cumulative over the long period of the pandemic, has not yet been fully investigated for Germany.

**Objectives:**

The aim of the study was to determine psychological burden as well as COVID-19-related experience and behavior patterns and to show how they changed during the different phases of the pandemic in Germany.

**Materials and methods:**

The Germany-wide online-based cross-sectional study (03/10–07/27/2020) included 22,961 people (convenience sample). Generalized anxiety (GAD-7), depression (PHQ-2), and psychological distress (DT) were collected, as well as COVID-19-related experiences and behavior patterns: *COVID-19-related fear, trust in governmental actions, subjective level of information, adherent safety behavior, *and *personal risk assessment for infection/severe course of illness*. The pandemic was retrospectively divided into five phases (initial, crisis, lockdown, reorientation, and new normality).

**Results:**

Compared to pre-COVID-19 reference values, GAD‑7, PHQ‑2, and DT levels were significantly elevated and persistent throughout the different phases of the pandemic. *COVID-19-related fear*, *information level*, *trust*, *safety behavior*, and the *risk assessment for infection/severe course of illness* showed, after initial strong increase, a strong decrease to partly below the initial value. Exceptions were constant *risk assessments of having a severe course of illness or dying of it*.

**Conclusions:**

The increased levels of psychological burden, which have persisted throughout all phases of the pandemic, illustrate the need for sustainable support services. Declining values over the duration of the pandemic in terms of trust in governmental actions and the feeling of being well informed underline the need for more targeted education.

## Hintergrund

Die COVID-19-Pandemie, die sich seit Dezember 2019 weltweit ausbreitet und seit Ende Februar 2020 auch Deutschland in Atem hält, betrifft alle Lebensbereiche. Der Ausbreitungsweg von Wuhan, China [1], über Südostasien, Europa und die restliche Welt [2] war nicht nur eine Belastungssituation für das weltweite medizinische Versorgungssystem, sondern schränkte auch das öffentliche Leben in erheblichem Maße ein mit Folgen für verschiedene weitere Systeme wie Familie, Wirtschaft, Bildung, Kultur.

Deutschland reagierte nach ersten Infektionen in Bayern, Baden-Württemberg und Nordrhein-Westfalen ab Anfang März mit einem schrittweisen Lockdown, der am 22.03.2020 mit der Feststellung einer epidemischen Lage von nationaler Tragweite (gem. § 5 Infektionsschutzgesetz [IfSG]) mit der Umsetzung des nationalen Pandemieplanes u. a. in einem bundesweiten Kontaktverbot mündete [3, 4]. Es stellte sich im Sommer eine „neue Normalität“ mit Lockerungen ein, welche jedoch ab Herbst mit ansteigenden Infektionszahlen wieder in partielle Lockdowns national und international übergingen [5].

Im Mai 2020 erklärte der Generaldirektor der Weltgesundheitsorganisation (WHO), Tedros Adhanom Ghebreyesus, dass der Einfluss der Pandemie auf die psychische Gesundheit der Menschen bereits höchst besorgniserregend sei [6]. Diese Beobachtung konnte seit Beginn der Pandemie auch wissenschaftlich untermauert werden [7]. Eine stetige Zunahme an Evidenz bezüglich psychischer Gesundheit und der COVID-19-Pandemie aus chinesischen und südostasiatischen Querschnittsstudien zeigte, dass nicht nur Mitarbeitende des Gesundheitswesens belastet waren [8, 9], sondern dass auch in der Allgemeinbevölkerung Symptome von generalisierter Angst, Depression und schlechter Schlafqualität zunahmen [10–12]. Diese Ergebnisse wurden auch in Europa reproduziert [13–17]. Auch die Rolle des Übermaßes an medialen Informationen in Bezug auf COVID-19 und des Vertrauens in Institutionen und Regierungen in Bezug auf die psychische Belastung der Bevölkerung rückte in den Untersuchungsschwerpunkt [18–21]. Es wurde deutlich, dass die Bevölkerung hohes Vertrauen in staatliche Institutionen und deren Informationen beibehielt [17, 22].

Die Maßnahmen in Deutschland erwiesen sich im hier eher „milden“ Pandemiegeschehen der „ersten Welle“ bis in den Sommer als erfolgreich. Es kam zu keiner Dekompensation des Gesundheitswesens und auch die Intensivversorgung war zu keinem Zeitpunkt kritisch [23]. Dennoch wurde hier bereits deutlich, dass die Allgemeinbevölkerung in diesem Zeitraum im Durchschnitt höhere Werte an generalisierter Angst, Depression und psychischem Distress aufwies als vor der Pandemie [13, 24].

Erste chinesische Beobachtungen zeigten anhaltend erhöhte psychische Belastungen, auch noch nach über einem Monat nach Pandemieausbruch [12]. Zur Belastung der deutschen Bevölkerung durch die verschiedenen Phasen existieren zwar Daten [17], jedoch sind Untersuchungen, die die verschiedenen Zeiträume des Pandemiegeschehens, wie z. B. die Phase des Lockdowns mit der Phase der Lockerung, vergleichen, für Europa und speziell Deutschland noch rar [25]. Diesbezügliche Kenntnisse sind aber wichtig, um die verschiedenen Belastungsmerkmale der Bevölkerung zu unterschiedlichen Zeitpunkten besser verstehen und diesen damit besser begegnen zu können, um z. B. Unterstützungsformate wie Informationsangebote, telefonische Beratung oder Onlineinterventionen lageangepasst zu entwickeln [26–31].

Ziel der Untersuchung war es, aus einer großen Stichprobe der deutschen erwachsenen Allgemeinbevölkerung bezüglich der psychischen Belastungsmerkmale während der anhaltenden Pandemie (Zeitraum März–Juli 2020) verschiedene Pandemiephasen zu definieren und zu vergleichen. Es sollte der Frage nachgegangen werden, wie sich die Pandemiephasen unterschieden hinsichtlich psychischer Belastungen und verschiedener Ausprägungen von COVID-19-bezogenen Erlebens- und Verhaltensweisen, wie z. B. Angst, Verhalten, Informiertheitslevel und subjektive Risikoeinschätzungen, an COVID-19 zu erkranken. Aus diesen Erkenntnissen und in Zusammenschau mit weiterer internationaler Literatur sollen existierende und ggf. notwendige Hilfsangebote mit den tatsächlich existierenden Bedürfnissen abgeglichen und diskutiert werden.

## Material und Methoden

### Studiendesign und Studienteilnehmende

Die deutschlandweite anonyme onlinebasierte Querschnittsstudie erfolgte über das Online-Umfrage-Softwareprogramm Unipark (Questback GmbH). Die Rekrutierung erfolgte über Berichte in regionalen und überregionalen Radio- und Fernsehbeiträgen. Offizielle Homepages (z. B. Kliniken, Kommunen) verwiesen auf die Untersuchung. Es erfolgte ebenso eine Rekrutierung via Social-Media-Kanäle (WhatsApp, Facebook, Instagram, Twitter) sowohl über private als auch öffentliche Accounts von Institutionen und Rundfunkeinrichtungen. Bei der Stichprobe handelt es sich somit um ein Convenience Sample, also eine willkürliche Stichprobe mit eingeschränkter Generalisierbarkeit. Eine mehrfache Teilnahme an der Befragung wurde technisch mithilfe eines IP-Adressenblocks nach erfolgter Teilnahme weitestgehend vermieden.

Im Erhebungszeitraum vom 10.03.2020 bis zum 27.07.2020 wurden 22.961 Personen erreicht, von 18.301 Teilnehmenden lagen komplette Datensätze vor (79,7 %). 16.918 (73,7 %) Teilnehmende gaben Deutschland als Wohnort an und waren mindestens 18 Jahre alt; sie wurden somit in die Auswertungen eingeschlossen.

Der Erhebungszeitraum wurde retrospektiv gemäß den Reaktionen des öffentlichen Gesundheitswesens auf die Virusausbreitung und den entsprechenden Maßgaben in 5 Phasen eingeteilt, die untereinander verglichen werden konnten. Eine Übersicht über diese Phasen und die dazugehörigen Ereignisse sind Tab. 1 zu entnehmen.

**Table 1:** 

Phase	Zeitraum	Ereignisse	Teilnehmendenzahl
**1 Anfangsphase**	10.03.–15.03.2020	Zunehmende Ausbreitung von COVID-19-Fällen in Bayern, Baden-Württemberg und Nordrhein-Westfalen; erste Absagen von Großveranstaltungen	*n* = 6535
**2 Krisenphase**	16.03–22.03.2020	Erste Ausgangssperren in Bayern bis zur deutschlandweiten Kontaktsperre	*n* = 4368
**3 Lockdownphase**	23.03.–14.04.2020	Lockdown des öffentlichen Lebens bis hin zum Beschluss erster Öffnungen im öffentlichen Einzelhandel	*n* = 2826
**4 Neuorientierungsphase**	15.04.–25.05.2020	Schrittweise weitere Lockerungen des öffentlichen Lebens bis hin zur Lockerung im Tourismussektor	*n* = 1634
**5 „neue Normalität“**	26.05.–27.07.2020	Weitestgehende Stabilität im Infektionsgeschehen und in öffentlichen Beschränkungen, sporadische Hotspots und lokale Lockdowns	*n* = 1555
*Gesamt*			*n* *=* *16.918*

### Messinstrumente

Die Befragung umfasste neben soziodemografischen Angaben folgende Inhalte:

#### Psychische Belastung

Zur Erhebung der psychischen Belastung wurden 3 validierte Instrumente verwendet. Zur Erhebung der generalisierten Angstsymptome wurde die deutsche Version des *GAD‑7* (*Generalized Anxiety Disorder‑7*, 7 Items, 4‑Punkt-Likert-Skala rangierend von 0 = überhaupt nicht bis 3 = beinahe jeden Tag; [32]) verwendet. Entsprechend vorangegangener deutscher Validierungsuntersuchungen des GAD‑7 [33] wurden Summenscores von ≥ 5, ≥ 10 und ≥ 15 als milde, moderate und schwere generalisierte Angstsymptome bewertet. Zur Erhebung von depressiven Symptomen wurde die deutsche Version des *PHQ‑2* (*Patient Health Questionnaire‑2, *2 Items, 4‑Punkt-Likert-Skala; 0 = überhaupt nicht bis 3 = beinahe jeden Tag; [34]) verwendet. Ein Summenscore von ≥ 3 weist hierbei auf eine Major Depression bzw. eine schwere depressive Symptomatik hin. Zur Erhebung von psychischem Distress wurde das *DT* (*Distressthermometer*, 1 Item, visuelle Analogskala von 0–10; 0 = kein psychischer Distress bis 10 = extremer psychischer Distress; [35]) verwendet. Werte, die ≥ 5 sind, weisen auf einen erhöhten psychischen Distress hin.

#### COVID-19-bezogene Erlebens- und Verhaltensweisen

Zur Erhebung der COVID-19-bezogenen Erlebens- und Verhaltensweisen wurden, entsprechend der jeweils aktuellen politischen Entwicklungen und im abteilungsinternen Expertenkonsensus, Items entwickelt zur Erhebung von:

- *COVID-19-bezogener Angst*,
- *Vertrauen in staatliche Maßnahmen in Bezug auf COVID-19*,
- *subjektivem Informiertheitslevel in Bezug auf COVID-19* und
- *adhärentem (staatlichen/wissenschaftlichen Empfehlungen folgendem) Sicherheitsverhalten in Bezug auf COVID-19* (Einhalten von Hygieneregeln).

Alle Fragen zu COVID-19-bezogenen Erlebens- und Verhaltensweisen wurden mit einer 7‑Punkt-Likert-Skala (1 = „stimme überhaupt nicht“ bis 7 = „stimme völlig zu“) erhoben.

*COVID-19-bezogene Angst* wurde mittels eines Items erfragt. *Vertrauen in staatliche Maßnahmen *und das *subjektive Informiertheitslevel *wurden mittels dreier Items erfragt. Das *adhärente Sicherheitsverhalten *wurde mit 4 Items erfragt.

Die Reliabilität für die 3 Skalen *Vertrauen in staatliche Maßnahmen*, *subjektives Informiertheitslevel* und *adhärentes Sicherheitsverhalten *wurden getestet. Cronbachs α diente hierbei als Maß der internen Konsistenz. Alle 3 Skalen zeigten hohe interne Konsistenz mit einem Cronbachs α = 0,825 bzw. Cronbachs α = 0,801 und Cronbachs α = 0,738 (vgl. auch [13, 16, 36]). Die Items zu den Skalen sind im Online-Zusatzmaterial (Tab. Z1) dargestellt.

#### Persönliche Risikoeinschätzungen für Ansteckung und Erkrankung

Zur *persönlichen Risikoeinschätzung *wurden 3 verschiedene Einschätzungen über 3 Items abgefragt: „Wie hoch schätzen Sie die Wahrscheinlichkeit ein, dass Sie an COVID-19 (Corona-Virus) erkranken?“ „Wenn Sie an COVID-19 (Corona-Virus) erkranken würden: Wie hoch schätzen Sie die Wahrscheinlichkeit ein, dass diese Erkrankung einen schweren Verlauf nimmt?“ „Wenn Sie an COVID-19 (Corona-Virus) erkranken würden: Wie hoch schätzen Sie die Wahrscheinlichkeit ein, dass Sie an dieser Erkrankung versterben?“ Die Teilnehmenden konnten über eine visuelle Analogskala eine Einschätzung von 0–100 % abgeben.

Das Ausfüllen des Fragebogens dauerte im Mittel 11 min 23 s.

### Statistische Auswertung

Eine lineare Regressionsanalyse wurde verwendet, um die Unterschiede zwischen den 5 Phasen zu ermitteln. Abhängige Variablen waren die psychische Belastung, gemessen mittels *GAD‑7, PHQ‑2* und DT, COVID-19-bezogenen Erlebens- und Verhaltensweisen sowie die *persönlichen Risikoeinschätzungen für Ansteckung/Erkrankung mit SARS-CoV-2/COVID-19*. Zunächst wurden die Zeitpunkte als kategoriale, treatmentcodierte unabhängige Variable hinzugefügt.

Da es sich um ein wiederholendes Querschnittsdesign handelte, könnten jedoch Kovariaten einen verzerrten Zeitverlauf erzeugen, z. B. durch eine Erhöhung von Angst in einem gewissen Zeitraum, welche jedoch lediglich der zufälligen oder systematischen (z. B. durch Verbreitungsdynamiken in Kommunikationskanälen) zeitlichen Anhäufung von Teilnehmenden geschuldet wäre. Um dem entgegenzuwirken, wurde die Regressionsanalyse gewählt. Diese ist bis zu einem gewissen Grad in der Lage, solche bestehenden Unterschiede in den Teilnehmendencharakteristika herauszurechnen, indem sie den Einfluss dieser Unterschiede in die Berechnung miteinbezieht. Der Regressionskoeffizient bildet somit den Einfluss des Prädiktors durch den Einschluss aller anderen Variablen ab.

Um nun den Einfluss der kategorialen Variable „Zeitraum“ möglichst unverzerrt darzustellen, wurden in der hier vorgestellten Analyse nicht nur die jeweiligen Phasen als unabhängige Variablen, sondern auch Alter, Geschlecht, Bildung, Beruf, Vorliegen einer psychischen Erkrankung, Vorliegen einer körperlichen Risikoerkrankung für einen schlechten COVID-19-Verlauf sowie die Wohnortgröße als Kovariaten betrachtet. Der zeitliche Verlauf der abhängigen Variablen wurde in den verwendeten Abbildungen als Rohmittelwert dargestellt. Um Unterschiede zwischen den Zeitpunkten zu ermitteln, wurden zudem konditionale Effekte für jede Regressionsanalyse ermittelt (R-Paket *emmeans*). Im Ergebnisteil werden globale F‑Tests berichtet, um Unterschiede in den Variablen zu verdeutlichen. Effektstärken wurden über Cohens *d *berechnet; hierbei war ein Cohens *d* um 0,2 ein kleiner, *d* um 0,5 ein mittlerer und *d* um 0,8 ein großer Effekt. Die konditionalen Effekte (Tab. Z2) ebenso eine ausführliche Auflistung der Regressionskoeffizienten (Tab. Z3) werden im Online-Zusatzmaterial dargestellt.

Aufgrund der sehr großen Stichprobengröße war davon auszugehen, dass sich eine Verletzung der Normalverteilungsannahme der Residuen nicht negativ auf die Regressionsschätzer auswirkt [37]. Auf Homoskedastizität wurde mit dem Breusch-Pagan-Test getestet. Im Falle einer Verletzung dieser Voraussetzung wurden zusätzlich robuste Regressionen berechnet (*lmrob* R‑Paket *robustbase*) siehe Online-Zusatzmaterial Tab. Z4, um zu überprüfen, ob die Schätzung verzerrt wurde. Die gesamte Analyse wurde mit R (Version 3.6.3) durchgeführt.

## Ergebnisse

### Stichprobenbeschreibung

Eine Übersicht über die in den Analysen verwendeten demografischen Daten, gesamt und aufgeschlüsselt nach Pandemiephasen, ist in Tab. 2 zu finden.

**Table 2:** 

		Erste Phase	Zweite Phase	Dritte Phase	Vierte Phase	Fünfte Phase	Gesamt
*n*		6535	4368	2826	1634	1555	16.918
*Geschlecht*	Weiblich	4715 (72,1)	3146 (72,0)	2005 (70,9)	989 (60,5)	929 (59,7)	11.784 (69,7)
	Männlich	1800 (27,5)	1211 (27,7)	808 (28,6)	638 (39,0)	620 (39,9)	5077 (30,0)
	Divers	20 (0,3)	11 (0,3)	13 (0,5)	7 (0,4)	6 (0,4)	57 (0,3)
*Alter*	18–24 Jahre	748 (1,4)	685 (15,7)	494 (17,5)	214 (13,1)	204 (13,1)	2345 (13,9)
	25–34 Jahre	1734 (26,5)	1129 (25,8)	634 (22,4)	308 (18,8)	310 (19,9)	4115 (24,3)
	35–44 Jahre	1601 (24,5)	935 (21,4)	639 (22,6)	350 (21,4)	364 (23,4)	3889 (23,0)
	45–54 Jahre	1271 (19,4)	767 (17,6)	550 (19,5)	330 (20,2)	320 (20,6)	3238 (19,1)
	55–64 Jahre	874 (13,4)	649 (14,9)	379 (13,4)	280 (17,1)	254 (16,3)	2436 (14,4)
	65–74 Jahre	255 (3,9)	180 (4,1)	113 (4,0)	123 (7,5)	84 (5,4)	755 (4,5)
	75+ Jahre	52 (0,8)	23 (0,5)	17 (0,6)	29 (1,8)	19 (1,2)	140 (0,8)
*Bildung*	Universitätsabschluss	2892 (44,3)	1901 (43,5)	1117 (39,5)	655 (40,1)	600 (38,6)	7165 (42,4)
	Abitur	2085 (31,9)	1459 (33,4)	977 (34,6)	503 (30,8)	459 (29,5)	5483 (32,4)
	Realschulabschluss	1175 (18,0)	739 (16,9)	536 (19,0)	351 (21,5)	372 (23,9)	3173 (18,8)
	Hauptschulabschluss	276 (4,2)	176 (4,0)	132 (4,7)	83 (5,1)	93 (6,0)	760 (4,5)
	Kein Abschluss	14 (0,2)	17 (0,4)	16 (0,6)	1 (0,1)	8 (0,5)	56 (0,3)
	Sonstige	93 (1,4)	76 (1,7)	48 (1,7)	41 (2,5)	23 (1,5)	281 (1,7)
*Gemeindegröße (Einwohnerzahl)*	>100.000	3872 (59,3)	2543 (58,2)	1360 (48,1)	776 (47,5)	626 (40,3)	9177 (54,2)
	Zwischen 20.000 und 100.000	1453 (22,2)	975 (22,3)	681 (24,1)	355 (21,7)	378 (24,3)	3842 (22,7)
	Zwischen 5000 und 20.000	645 (9,9)	434 (9,9)	393 (13,9)	227 (13,9)	246 (15,8)	1945 (11,5)
	Unter 5000	565 (8,6)	416 (9,5)	392 (13,9)	276 (16,9)	305 (19,6)	1954 (11,5)
*Beruf*	Andere	3701 (56,6)	2639 (60,4)	1815 (64,2)	1053 (64,4)	1005 (64,7)	10.213 (60,4)
	Arbeitslos	531 (8,1)	510 (11,7)	287 (10,2)	243 (14,9)	194 (12,5)	1765 (10,4)
	Arzt/Ärztin	357 (5,5)	98 (2,2)	62 (2,2)	36 (2,2)	35 (2,3)	588 (3,5)
	Pflegekraft	955 (14,6)	412 (9,4)	210 (7,4)	107 (6,5)	114 (7,3)	1798 (10,6)
	Rettungsdienst/Feuerwehr/Polizei	212 (3,2)	62 (1,4)	51 (1,8)	21 (1,3)	23 (1,5)	369 (2,2)
	Schüler*innen/Studierende	778 (11,9)	647 (14,8)	401 (14,2)	174 (10,6)	183 (11,8)	2183 (12,9)
*Psychische Erkrankung*	Vorliegend	554 (8,5)	853 (19,5)	441 (15,6)	162 (9,9)	140 (9,0)	2150 (12,7)
*COVID-19-spezifische Risikoerkrankung*	Vorliegend	1486 (22,7)	958 (21,9)	571 (20,2)	383 (23,4)	344 (22,1)	3742 (22,1)

### Generalisierte Angst, Depressivität und psychischer Distress

**Figure 1:**
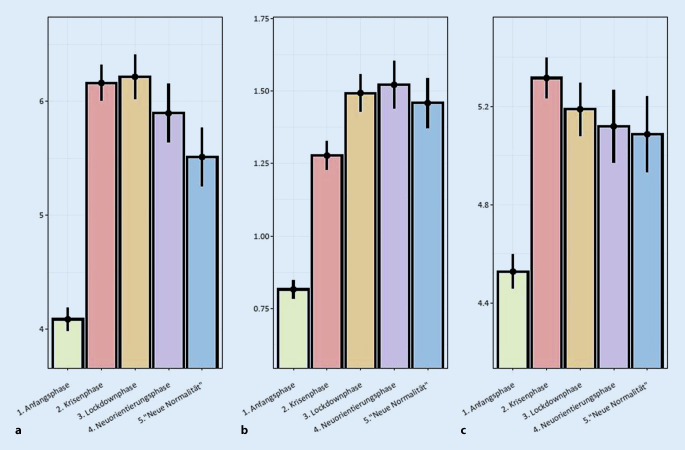

Beim GAD‑7 (globaler F‑Test: F(4, 16.888) = 124,62, *p* < 0,001) ergaben sich Unterschiede zwischen Phase 1 und allen anderen Phasen mit *p* < 0,001 (alle Effektgrößen d > 0,32; Abb. 1). Es ergab sich ferner ein signifikanter Unterschied zwischen Phase 2 und 4 mit *p* = 0,043, jedoch mit einer geringen Effektstärke von d = 0,082. Die Werte des PHQ‑2 erhöhten sich konstant bis Phase 4 (globaler F‑Test: F(4, 16.888) = 130.350, *p* < 0,001). Hier fanden sich signifikante Unterschiede zwischen Phase 1 und den restlichen Phasen (alle *p* < 0,001, alle d > 0,18), Phase 2 und den darauffolgenden Phasen (alle *p* < 0,002, alle d > 0,189) sowie Phase 3 und 4 (*p* = 0,005, d = 0,108). Das DT hingegen wies nach der Erhöhung von Phase 1 zu Phase 2 ein konstantes Level auf (globaler F‑Test = F(4, 16.888) = 46.101, *p* < 0,001). Es ergaben sich lediglich signifikante Unterschiede zwischen Phase 1 und den darauffolgenden Phasen (alle *p* < 0,001, alle d > 0,167).

Aus Tab. 3 sind die proportionalen Anteile der Teilnehmenden pro Phase zu entnehmen, die über den jeweiligen Cut-offs (Grenzwerten) für GAD‑7, PHQ‑2 und des DT liegen. Die Ergebnisse aller 7 Regressionsanalysen sind im Online-Zusatzmaterial (Tab. Z4) dargestellt.

**Table 3:** 

		1 Anfangsphase (*n* = 6535)		2 Krisenphase (*n* = 4368)		3 Lockdownphase (*n* = 2826)		4 Neuorientierungsphase (*n* = 1634)		5 „neue Normalität“ (*n* = 1555)	
Skala	Cut-off	*n*	%	*n*	%	*n*	%	*n*	%	*n*	%
*GAD‑7*	Keine Auffälligkeit (<5 Punkte)	4253	65	2081	48	1331	47	816	50	816	52
	Leicht (≥5 <10 Punkte)	1640	25	1327	30	864	31	471	29	426	27
	Moderat (≥10 <15 Punkte)	414	06	535	12	352	12	206	13	205	13
	Schwer (≥15 Punkte)	228	03	425	10	279	10	141	9	108	7
*PHQ‑2*	Unter dem Cut-off für Major Depression (<3)	6019	92	3622	83	2235	79	1274	78	1222	79
	Über dem Cut-off für Major Depression (≥3)	516	08	746	17	591	21	360	22	333	21
*DT*	Kein erhöhter Distress (<5)	3173	49	1611	37	1119	40	683	42	650	42
	Erhöhter Distress (≥5)	3362	51	2757	63	1707	60	951	58	905	58

*GAD‑7* zur Messung von Symptomen generalisierter Angst (Generalized Anxiety Disorder‑7, 7 Items, 4‑Punkt-Likert-Skala rangierend von 0 = überhaupt nicht bis 3 = beinahe jeden Tag), *PHQ‑2* zur Messung von depressiven Symptomen (Patient Health Questionnaire‑2, 2 Items, 4‑Punkt-Likert-Skala rangierend 0 = überhaupt nicht bis 3 = beinahe jeden Tag), *DT* zur Messung von Distress (Distressthermometer, 1 Item, visuelle Analogskala von 0–10, rangierend von 0 = kein Distress bis 10 = extremer Distress)

### COVID-19-bezogene Erlebens- und Verhaltensweisen (Angst, Vertrauen und Information)

**Figure 2:**
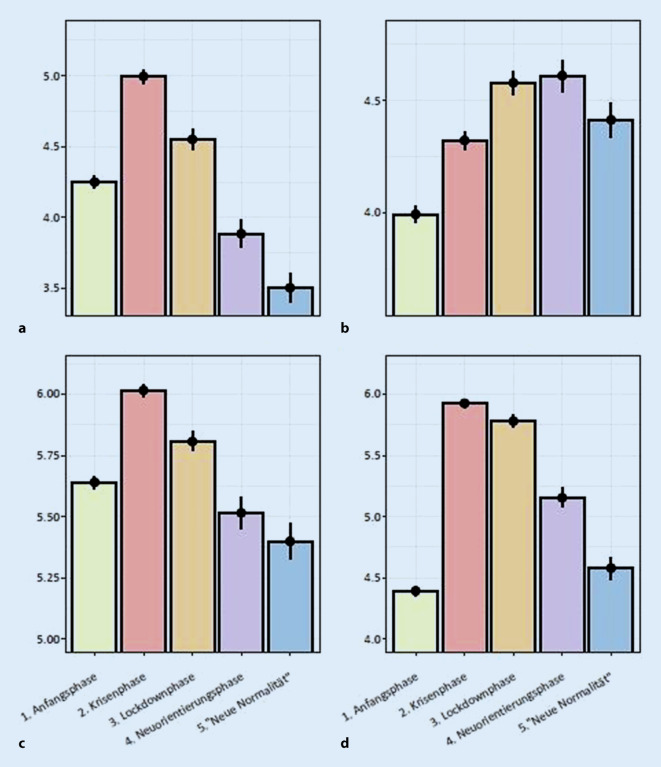

In den COVID-19-bezogenen Erlebens- und Verhaltensweisen zeigte sich ein zunächst steigender, dann jedoch wieder abfallender Trend sowohl in der *COVID-19-bezogenen Angst*, im *Vertrauen in staatliche Maßnahmen,* im *subjektiven Informiertheitslevel *und im *adhärenten Sicherheitsverhalten* (Abb. 2). Bei der *COVID-19-bezogenen Angst* waren alle paarweisen Vergleiche mit einem *p* < 0,001 signifikant (F(4, 16.888) = 234,32, *p* < 0,001), wobei sich die größten Unterschiede aus den Vergleichen zwischen Phase 1 und 2 (mit d = 0,395), Phase 2 und 4 (d = 0,592) sowie Phase 2 und 5 (d = 0,789) ergaben. Auch das *Vertrauen in staatliche Maßnahmen *erhöhte sich zunächst über die Zeit (F(4, 16.888) = 124.582, *p* < 0,001): Die Phasen 1 und 2 unterschieden sich signifikant von allen darauffolgenden (alle *p* ≤ 0,027, Phase 2 vs. 5 d = 0,088, alle anderen Vergleiche d > 0,198). Phase 3 und 4 unterschieden sich nicht (*p* = 1). In Phase 5 verringerte sich das Vertrauen wieder im Vergleich zu Phase 3 und 4 (beide *p* ≤ 0,016), jedoch nur leicht (beide d = 0,110). Das *subjektive Informiertheitslevel *stieg stark zu Phase 2 an, fiel dann jedoch wieder ab (F(4, 16.888) = 132.570, *p* < 0,001). Auch hier ergaben sich die stärksten paarweisen Unterschiede zwischen Phase 1 und 2 (*p* < 0,001, d = 0,352) sowie der Phase 2 und 4 (*p* < 0,001, d = 0,451) und Phase 2 und 5 (*p* < 0,001, d = 0,533). Keine Unterschiede ergaben sich zwischen den letzten beiden Phasen (*p* = 0,141, d = 0,082). Das *adhärente Sicherheitsverhalten *zeigte einen starken Anstieg von Phase 1 zu 2, die Werte sanken jedoch über die Zeit wieder ab (F(4, 16.888) = 951,53, *p* < 0,001). Alle paarweisen Vergleiche wurden signifikant (mit *p* ≤ 0,008), jedoch ergaben sich die stärksten Unterschiede zwischen Phase 1 und 2 (d = 1,077).

### Persönliche Risikoeinschätzung für Ansteckung und Erkrankung

**Figure 3:**
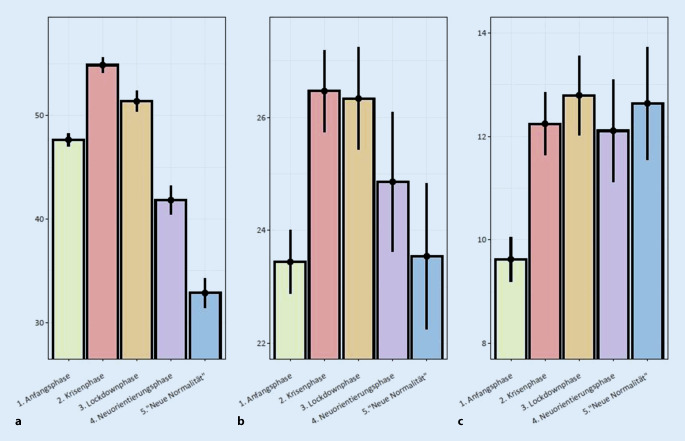

Analog zur COVID-19-bezogenen Angst fand sich auch in der *persönlichen Risikoeinschätzung für eine Ansteckung mit SARS-CoV‑2* ein zum Zeitpunkt des primären Lockdowns ansteigendes, dann jedoch abflachendes Verlaufsmuster (Abb. 3, F(4, 16.888) = 218,470). In allen paarweisen Vergleichen fanden sich signifikante Unterschiede (*p* < 0,001), wobei die stärksten Unterschiede erneut zwischen Phase 1 und 2, Phase 2 und 4 sowie Phase 2 und 5 zu finden waren. Der Unterschied zwischen Phase 2 und 3 fiel gering aus. Ein umgekehrt-u-förmiger Verlauf, jedoch mit sehr geringen Effektgrößen, fand sich auch in der *persönlichen Risikoeinschätzung für einen schweren Verlauf bei Erkrankung mit COVID-19* (F(4, 16.888) = 14,407, *p* < 0,001). Es fand sich eine Erhöhung von Phase 1 zu Phase 2 und 3 (in beiden Fällen < 0,001, d = 0,11 und d = 0,113), dann wieder eine Verringerung der Risikoeinschätzung von Phase 2 und 3 zu Phase 4 und 5 (alle *p* ≤ 0,002, alle d > 0,11). Der Unterschied zwischen Phase 1 und 5 war hierbei nicht signifikant (*p* = 0,658).

Letztlich wurde beobachtet, dass die *persönliche Risikoeinschätzung, an COVID-19 zu versterben,* in allen Phasen mit etwa 20 % bewertet wurde. Zwischen den Phasen ergaben sich auch hier Unterschiede, die jedoch gering waren: Die Risikoeinschätzung stieg zu Phase 2 an (F(4, 16.888) = 16,464, *p* < 0,001; Phase 1 vs. Phase 2: *p* < 0,001, d = 0,121) und blieb weitestgehend erhöht. In Phase 4 war die Risikoeinschätzung leicht verringert, der Unterschied erreichte das Signifikanzkriterium jedoch nicht (*p* = 0,062).

## Diskussion

Mit dieser bislang größten deutschlandweiten Studie zu psychischer Belastung im Kontext der COVID-19-Pandemie zeigten sich generalisierte Angst, depressive Symptomatik und psychischer Distress seit Beginn der Pandemie im Erhebungszeitraum relativ stabil im Durchschnitt auf erhöhtem Niveau, was sich auch mit Erkenntnissen eines longitudinalen Designs von Bendau et al. deckt [25].

Die höchsten Ausprägungen der generalisierten Angst ließen sich hierbei in der Phase 3 „Lockdown“ beobachten mit 31 % der Teilnehmenden mit leichten, 12 % mit moderaten und 10 % mit schweren Symptomen einer generalisierten Angst. Dies ist besonders bemerkenswert, da die Ausprägungen von moderater bzw. schwerer generalisierter Angstsymptomatik in normativen Stichproben vor der COVID-19-Pandemie bei 6,0 % bzw. 5,9 % (moderat) und 1 % bzw. 1,2 % (schwer) lagen [32, 33]. Dies bedeutet, dass in dieser psychisch belastenden Phase generalisierte Angst zweifach bzw. zehnfach erhöht war.

Symptome einer Major Depression waren beinahe über die gesamte Zeit der Pandemie, von Phase 2 „Krisenphase“ bis Phase 5 „neue Normalität“ bei 17–22 % der Teilnehmenden zu finden, im Vergleich zu einer normativen Prä-COVID-19-Stichprobe mit einer Prävalenz von 5,6 % [38].

Psychischer Distress, der in der Phase 5 „neue Normalität“ einen leichten Abwärtstrend zeigte, aber auch hier deutlich vom Ausgangswert entfernt war, zeigte in der „Krisenphase“ höchste Ausprägungen mit 63 % der Teilnehmenden, die über dem Cut-off lagen. Auch dies steht im Vergleich zu Prä-COVID-19-Stichproben mit einer Prävalenz von 39 % [35].

Währenddessen verhielten sich *COVID-19-bezogene Angst, Vertrauen in staatliche Maßnahmen in Bezug auf COVID-19,* das* subjektive Informiertheitslevel in Bezug auf COVID-19* und das *adhärente Sicherheitsverhalten in Bezug auf COVID-19 *übergreifend mit einem starken Anstieg zur „Krisenphase“ und einem deutlichen Abfall, z. T. unter den Ausgangswert in der Phase der „neuen Normalität“.

Die *persönliche Risikoeinschätzung für Ansteckung/Erkrankung mit SARS-CoV-2/COVID-19* zeigte ein heterogenes Bild. Die persönliche Risikoeinschätzung, sich mit SARS-CoV‑2 zu infizieren, ist seit Beginn der Pandemie nach einem Höhepunkt während der „Krisenphase“ deutlich abgesunken, zum Schluss sogar deutlich unter den Ausgangswert, mit im Mittel nur noch geschätzten 32,86 % Wahrscheinlichkeit. Hingegen hielt sich die persönliche Risikoeinschätzung, potenziell einen schweren Verlauf von COVID-19 zu haben bzw. daran zu versterben, seit Beginn der Pandemie relativ konstant. Das ist im Lichte abnehmenden adhärenten Verhaltens überraschend, da man bei konstanter Einschätzung eines schweren Verlaufes auch konstant dazu passendes Verhalten erwarten würde, auch im Hinblick auf die Risikoeinschätzung, an COVID-19 zu versterben. Diese zeigte zuletzt sogar leicht steigende Tendenz.

Deutschland zeigte sich im ersten dreiviertel Jahr der anhaltenden COVID-19-Pandemie als eines der robustesten, am wenigsten betroffenen Länder. Versorgungsketten und Krankenhäuser waren überwiegend stabil, die Gefahr einer systematischen Überlastung der bundesweiten bzw. regionalen Intensivkapazitäten war zu keinem Zeitpunkt wahrscheinlich [23]. Das Ausbruchsgeschehen anhand der tagesaktuellen Zahlen des Robert Koch-Instituts (RKI) [39] war trotz zunächst exponentiellen Wachstums kontrolliert. Dennoch traf die COVID-19-Pandemie den Wirtschaftsstandort Deutschland hart. In den Konjunkturdaten des 2. Quartals 2020 zeigte sich ein Einbruch des Bruttoinlandsprodukts um 10,1 % im Vergleich zum Vorquartal – der stärkste Rückgang seit Beginn der Berechnungen 1970 [40, 41]. Ein Anstieg der Arbeitslosenquote um lediglich 0,1 % auf 6,3 % im Juli 2020 wurde durch den massiven Einsatz von Kurzarbeit erreicht [42]. Diese Faktoren gilt es zu bedenken, wenn die psychische Belastung im Sinne von generalisierter Angst, Depressionen und psychischem Distress der Bevölkerung im Kontext der Pandemie betrachtet wird. Trotz einer schrittweisen Rückkehr in eine „neue Normalität“ stellte diese offensichtlich anhaltend eine solche Belastung dar, dass Menschen mit erhöhter Angst, Depression und Distress reagierten.

Dass diese anhaltenden psychischen Belastungen nicht gleichzusetzen sind mit der Angst vor COVID-19 selbst, konnte in dieser Studie verdeutlicht werden und deckt sich mit den Ergebnissen anderer Studiengruppen, die ebenfalls ein Auseinanderdriften von anhaltender psychischer Belastung und COVID-19-bezogener Angst beobachten konnten [25]. Während die allgemeinen psychischen Belastungen wie generalisierte Angst, depressive Symptomatik und psychischer Distress erhöht blieben, zeigt die COVID-19-bezogene Angst, einer klassischen Habituationskurve gleich, einen starken Anstieg von der „Anfangsphase“ in die „Krisenphase“, in der sie auch ihren Höhepunkt erreichte. Danach folgte bis zur „neuen Normalität“ ein stetiger Abfall deutlich unter den Ausgangswert. Wird dies im Zusammenhang mit zunehmend nachlassender Anwendung von adhärentem Sicherheitsverhalten gesehen, dann entsteht der Eindruck, dass die COVID-19-bezogene Angst nicht als dysfunktionale Angst zu verstehen ist, sondern als eine Angst, die darin unterstützt, gefahrenadaptiertes Verhalten aufrecht zu halten. Solche Zusammenhänge wurden bereits in der H1N1-Epidemie in Hongkong bemerkt, die in 10 Querschnittsstudien einen Zusammenhang mit risikospezifischer Furcht (hier H1N1) und der Durchführung von Schutzverhalten beschrieben [43]. Diese nachlassende Furcht spiegelt sich vermutlich auch in den, ebenfalls nach der Krisenphase stark fallenden, persönlichen Risikoeinschätzungen, sich mit SARS-CoV‑2 zu infizieren, wider. Dies ist erstaunlich, da die Wahrscheinlichkeit, einen schweren Verlauf von COVID-19 zu erleben oder gar daran zu versterben, relativ konstant hoch über die gesamte Zeit eingeschätzt wurde. Insbesondere die Fatalitätseinschätzung mit zuletzt immer über 12 % scheint hierbei bemerkenswert, wo doch aktuelle europäische Daten von einer Case-Fatality-Rate (CFR) von 6,9 % ausgehen und von einer Infection-Fatality-Rate (IFR) von 0,1 % [44, 45]. Man schätzte also die Chance zu sterben anhaltend höher ein, als die Evidenz es vermuten lässt. Die Gefahr, sich überhaupt zu infizieren, wurde jedoch trotz steigender Fallzahlen weltweit [45] als zunehmend unwahrscheinlicher bewertet.

In dieses Bild passt auch der erst stark zu-, dann abnehmende Verlauf des subjektiven Informiertheitslevels in Bezug auf COVID-19. Wichtig zu erwähnen ist hierbei, dass die Daten auf ein im Mittel gutes gefühltes Informiertheitslevel hinweisen, was auch andere Studienergebnisse bestätigt [17, 22, 46]. Auch hier zeigte sich der Peak des subjektiven Informiertheitslevels in der „Krisenphase“ mit einem anschließenden Abfall unter den Ausgangswert. Die Zeitschrift *Lancet Infectious Disease* nahm im Juli 2020 Stellung zur „COVID-19 Infodemic“, nachdem der Generaldirektor der WHO die COVID-19-Pandemie als eine solche bezeichnet hatte [47]. Insbesondere Menschen mit geringer Gesundheitskompetenz berichteten über ein geringeres Gefühl von Informiertheit [46]. Chinesische Untersuchungen zeigten, dass je mehr jemand den medialen Informationsfluten ausgesetzt ist, desto mehr Distresserleben entsteht [48]. Auf der anderen Seite konnte gezeigt werden, dass ein höheres Gefühl von Informiertheit mit weniger generalisierter Angst, sowohl bei medizinischem Fachpersonal als auch in der Allgemeinbevölkerung, assoziiert ist [16].

Die Relevanz von vertrauensstiftenden Maßnahmen in Bezug auf politische Entscheidungen zur COVID-19-Ausbruchswelle für Deutschland konnte bereits bei Teufel et al. untersucht werden [49]. Hierbei war in einem Ausschnitt vom Beginn des Pandemiegeschehens beobachtet worden, wie die Adressierung der Bundeskanzlerin Angela Merkel an die Bevölkerung mit einer kurzzeitigen Reduktion der psychischen Belastung, insbesondere von Ängsten, zeitlich zusammenfiel. Australische Untersuchungen konnten zeigen, dass Menschen mit höherem Vertrauen in staatliche Maßnahmen sich eher an „Vermeidungsverhalten“, also adhärentes Sicherheitsverhalten, hielten als jene mit weniger Vertrauen [20]. Neuseeländische Untersuchungen zeigten, dass Menschen während der „Lockdownphase“ mehr Vertrauen in die Politik hatten, sich allerdings auch psychisch belasteter fühlten als zuvor [21]. Dies betont noch einmal die Bedeutung von klaren staatlichen Maßnahmen und gleichzeitiger transparenter Kommunikation, um einerseits psychische Belastungen zu reduzieren und andererseits situationsangemessenes Verhalten zu gewährleisten. In Bezug auf Kommunikation und Information ist allerdings zu bedenken, dass man bei dieser Diskussion die verschiedenen Medienformate differenzieren muss. Während offizielle Informationen ggf. eher beruhigen, könnten z. B. aufgebauschte Informationen in sozialen Medien die Ängste tendenziell eher erhöhen, wie Bendau et al. zeigten [50].

Bei der Beurteilung der Studie müssen Limitationen berücksichtigt werden. Die erhobenen Daten entstammen einem Querschnittsstudiendesign, kausale Schlussfolgerungen aus den Daten waren nicht oder nur eingeschränkt möglich. So besteht die Schwierigkeit der residualen Konfundierung, denn eine Regressionsanalyse kann lediglich solche Variablen einbeziehen, welche auch erhoben wurden. Um dieses Problem zu umgehen, wäre ein Zeitreihendesign sinnhaft, was sicher im weiteren Verlauf der Pandemie erwogen werden sollte. Darüber hinaus wurden die Daten über eine Onlineumfrage erfasst, die über digitale und analoge Kanäle verbreitet wurde. Aus diesem Grund sollte immer die Möglichkeit einer Selektionsverzerrung in Betracht gezogen werden. Eine solche Verzerrung kann auch in Kombination mit Verfahren zur Überprüfung vorliegender Assoziationen zu irreführenden Ergebnissen (siehe z. B. Collider-Bias, [51]) führen. Bedingt durch die Notwendigkeit, eine neuartige Situation ohne zuvor validierte Instrumente schnell zu erfassen, muss bemerkt werden, dass die COVID-19-bezogenen Erlebens- und Verhaltensweisen, nämlich *COVID-19-bezogene Angst, Vertrauen in staatliche Maßnahmen,* das* subjektive Informiertheitslevel *und das *adhärente Sicherheitsverhalten*, sowie die *persönliche Risikoeinschätzung für Ansteckung/Erkrankung mit SARS-CoV-2/COVID-19,* nur post hoc validiert werden konnten. Die Post-hoc-Validierung der Skalen zeigte allerdings eine hohe interne Konsistenz.

Zusammenfassend wird aus der vorliegenden Untersuchung klar, dass in Deutschland während der COVID-19-Pandemie im Untersuchungszeitraum deutlich erhöhte Werte für psychische Belastungen zu finden sind. Diese psychischen Belastungen hielten an, auch wenn die COVID-19-bezogene Furcht bereits nachließ, habituierte und eine „neue Normalität“ eintrat. Gleichzeitig fielen das subjektive Informiertheitslevel, Vertrauen in staatliche Maßnahmen und die Anwendung von adhärentem Sicherheitsverhalten im Verlauf der Pandemie stark ab. Dies lässt folgenden Schluss zu: Es ist wichtig, trotz Rückkehr zur Normalität, Menschen, insbesondere Risikogruppen, einen stabilen Zugang zu psychischen Unterstützungsprogrammen sowohl online als auch offline zu gewährleisten [21, 26–28].

Bei Bäuerle et al. [13] konnte hierbei bereits gezeigt werden, welche vulnerablen Gruppen besonders mit erhöhter generalisierter Angst, Depression und psychologischem Distress belastet waren: Vor allem Frauen zeigten mehr generalisierte Angst als Männer über alle Ausprägungsgrade, sie zeigten mehr Symptome einer Major Depression und erhöhten psychischen Distress. Frauen zeigten auch höhere COVID-19-bezogene Angst. Jüngere Menschen zeigten die höchsten Werte für generalisierte Angst in allen Ausprägungsgraden, ebenso zeigte diese Gruppe die höchste Ausprägung an Majordepression. Den höchsten psychologischen Distress hingegen zeigten Menschen im mittleren Alter. Die Altersgruppe < 65 Jahre zeigte am meisten COVID-19-bezogene Angst.

Verschiedene Angebote, gerichtet an die unterschiedlichen Bedürfnisse von Geschlecht und Altersgruppen, aber auch an die verschiedenen Phasen der Pandemie, gilt es, zu entwickeln und längerfristig in Anwendung zu bringen. Zudem scheint es von zentraler Bedeutung, dass konsequente Information und eine Einbeziehung in politische Maßnahmen durch Entscheidungsträger in Behörden und Politik erfolgen müssen, um konsequent adhärentes Verhalten zu gewährleisten, das notwendig ist, um die pandemische Situation im Griff zu behalten.

## Supplementary Information




