# Supplementary material for: Change in psychological burden during the COVID-19 pandemic in Germany: fears, individual behavior, and the relevance of information and trust in governmental institutions
Source: Bundesgesundheitsblatt Gesundheitsforschung Gesundheitsschutz. 2021 Jan 22;64(3):322–33. [Article in German] doi: 10.1007/s00103-021-03278-0 (PMC7821178; doi:10.1007/s00103-021-03278-0)
Supplement: Supplementary file 1 [file 103_2021_3278_MOESM1_ESM.pdf]

Bundesgesundheitsblatt 3/2021

Elektronisches Zusatzmaterial zum Beitrag:

Veränderung der psychischen Belastung in der COVID-19-Pandemie in Deutschland: Ängste, individuelles Verhalten und die Relevanz von Information sowie Vertrauen in Behörden

Eva-Maria Skoda[1], Anke Spura[2], Freia De Bock[2], Adam Schweda[1], Nora Dörrie[1], Madeleine Fink[1], Venja Musche[1], Benjamin Weismüller[1], Anke Benecke[1], Hannah Kohler[1], Florian Junne[3], Johanna Graf[3], Alexander Bäuerle[1], Martin Teufel[1]

[1]Klinik für Psychosomatische Medizin und Psychotherapie, Universität Duisburg-Essen, LVR-Klinikum Essen, Essen, Deutschland.
[2]Bundeszentrale für gesundheitliche Aufklärung (BZgA), Köln, Deutschland.
[3]Abteilung für Psychosomatische Medizin und Psychotherapie, Universität Tübingen, Medizinische Universitätsklinik, Tübingen, Deutschland.

Korrespondenzadresse
Dr. Eva-Maria Skoda
Universität Duisburg-Essen
Klinik für Psychosomatische Medizin und Psychotherapie
LVR-Klinikum Essen
Virchowstr. 174
45147 Essen
eva-maria.skoda@uni-due.de

Inhalt:

Messinstrumente
Tabelle Z1: Items zu den Skalen der COVID-19 bezogene Erlebens- und Verhaltensweisen

Regressionstabellen
Konditionale Effekte der Regressionsanalysen
Robuste Regressionen
Referenzen
Tabelle Z2: Konditionale Effekte der Regressionsanalysen
Tabelle Z3: Regressionskoeffizienten
Tabelle Z4: Robuste Regressionen

Messinstrumente

Tab. Z1: Items zu den Skalen der COVID-19 bezogene Erlebens- und Verhaltensweisen

SKALENNAME	ITEMS
COVID-19-BEZOGENE ANGST	1. „COVID-19 (Corona-Virus) beunruhigt mich“
VERTRAUEN IN STAATLICHE MAßNAHMEN IN BEZUG AUF COVID-19	1. „Ich denke, Deutschland ist gut auf COVID-19 (Corona-Virus) vorbereitet.“<br>2. „Ich denke, es werden alle staatlichen Maßnahmen ergriffen, um COVID-19 (Corona-Virus) zu bekämpfen.“<br>3. „Ich habe Vertrauen in das staatliche System in Deutschland.“
SUBJEKTIVES INFORMIERTHEITSLEVEL IN BEZUG AUF COVID-19	1. „Ich fühle mich über COVID-19 (Corona-Virus) informiert.“<br>2. „Ich fühle mich über Maßnahmen zur Vermeidung einer Ansteckung mit COVID-19 (Corona -Virus) informiert.“<br>3. „Ich verstehe die Hinweise der Gesundheitsbehörden hinsichtlich COVID-19 (Corona-Virus).“
ADHÄRENTES (STAATLICHEN/WISSENSCHAFTLICHEN EMPFEHLUNGEN FOLGENDES) SICHERHEITSVERHALTEN IN BEZUG AUF COVID-19	„Seit Ausbruch von COVID-19 (Corona-Virus) in Europa…“<br>1. „… wasche/desinfiziere ich mir häufiger die Hände.“<br>2. „…vermeide ich vermehrt öffentliche Plätze/Veranstaltungen“<br>3. „…vermeide ich vermehrt öffentlichen Nahverkehr (U-Bahn, Straßenbahn, Bus, Zug).“<br>4. „… habe ich meine Reise-/Urlaubspläne geändert oder würde Sie ändern, wenn ich etwas geplant hätte.“

Regressionstabellen

Konditionale Effekte der Regressionsanalysen

Um genau zu untersuchen, wie sich der Verlauf unserer gemessenen Konstrukte verhält, errechneten wir konditionale / marginale Effekte für die kategoriale Variable „Phase“ für jedes unserer zehn Regressionsmodelle. Die paarweisen Vergleiche, inklusive 95%-Konfidenzintervalle, p-Werte und Cohens d finden sich in den Tabellen Z2.

Robuste Regressionen

Aufgrund der Verletzung der Annahme der Homoskedastizität ist davon auszugehen, dass die Schätzer der linearen Regressionanalysen einem systematischen Bias unterliegen. Die Ergebnisse werden entsprechend um eine Neudarstellung der Analysen mittels heteroskedastizitäts-robuster Regression ergänzt, welche mit dem R Befehl vcovHC aus dem Paket sandwich (Zelleis, 2004) errechnet wurde. Die Ergebnisse sind in den Tabellen Z3a bis Z3c zu finden. Die Ergebnisse unterscheiden sich kaum von denen der ordinary least squares regression, welche in Tab. Z2 aufgeführt sind.

Referenz

Zeileis, A. (2004). Econometric Computing with HC and HAC Covariance Matrix Estimators. *Journal of Statistical Software, 11*(10), 1 - 17. doi:http://dx.doi.org/10.18637/jss.v011.i10

Tab. Z2: Konditionale Effekte der Regressionsanalysen

	Tabelle 2a: Generalisierte Angst (GAD-7)							
Vergleich	Schätzer / Differenz	Untere 95%-CI-Grenze	Obere 95%-CI-Grenze	Standardfehler	df	t-Ratio	p-Wert	d
erste Phase - zweite Phase	-1,492	-1,734	-1,250	0,089	16888,000	-16,799	0,000	-0,334
erste Phase - dritte Phase	-1,699	-1,978	-1,420	0,102	16888,000	-16,629	0,000	-0,380
erste Phase - vierte Phase	-1,856	-2,199	-1,514	0,126	16888,000	-14,782	0,000	-0,415
erste Phase - fünfte Phase	-1,454	-1,805	-1,104	0,128	16888,000	-11,334	0,000	-0,325
zweite Phase - dritte Phase	-0,207	-0,503	0,089	0,108	16888,000	-1,908	0,313	-0,046
zweite Phase - vierte Phase	-0,364	-0,722	-0,007	0,131	16888,000	-2,779	0,043	-0,081
zweite Phase - fünfte Phase	0,038	-0,328	0,403	0,134	16888,000	0,280	0,999	0,008
dritte Phase - vierte Phase	-0,157	-0,538	0,224	0,140	16888,000	-1,126	0,793	-0,035
dritte Phase - fünfte Phase	0,244	-0,143	0,632	0,142	16888,000	1,722	0,420	0,055
vierte Phase - fünfte Phase	0,402	-0,031	0,835	0,159	16888,000	2,532	0,084	0,090

	Tabelle 2b: Depressive Symptome (PHQ-2)							
Vergleich	Schätzer / Differenz	Untere 95%-CI-Grenze	Obere 95%-CI-Grenze	Standardfehler	df	t-Ratio	p-Wert	d
erste Phase - zweite Phase	-0,252	-0,328	-0,176	0,028	16888,000	-9,017	0,000	-0,179
erste Phase - dritte Phase	-0,518	-0,605	-0,430	0,032	16888,000	-16,101	0,000	-0,368
erste Phase - vierte Phase	-0,671	-0,778	-0,563	0,040	16888,000	-16,975	0,000	-0,477
erste Phase - fünfte Phase	-0,608	-0,718	-0,498	0,040	16888,000	-15,051	0,000	-0,432
zweite Phase - dritte Phase	-0,266	-0,359	-0,172	0,034	16888,000	-7,782	0,000	-0,189
zweite Phase - vierte Phase	-0,419	-0,531	-0,306	0,041	16888,000	-10,153	0,000	-0,298
zweite Phase - fünfte Phase	-0,356	-0,471	-0,241	0,042	16888,000	-8,445	0,000	-0,253
dritte Phase - vierte Phase	-0,153	-0,273	-0,033	0,044	16888,000	-3,484	0,005	-0,109
dritte Phase - fünfte Phase	-0,090	-0,212	0,032	0,045	16888,000	-2,017	0,258	-0,064
vierte Phase - fünfte Phase	0,063	-0,073	0,199	0,050	16888,000	1,262	0,715	0,045

	Tabelle 2c: Distress-Thermometer							
Vergleich	Schätzer / Differenz	Untere 95%-CI-Grenze	Obere 95%-CI-Grenze	Standardfehler	df	t-Ratio	p-Wert	d
erste Phase - zweite Phase	-0,615	-0,766	-0,465	0,055	16888,000	-11,142	0,000	-0,221
erste Phase - dritte Phase	-0,545	-0,718	-0,371	0,064	16888,000	-8,575	0,000	-0,196
erste Phase - vierte Phase	-0,679	-0,892	-0,466	0,078	16888,000	-8,697	0,000	-0,244
erste Phase - fünfte Phase	-0,622	-0,839	-0,404	0,080	16888,000	-7,795	0,000	-0,224
zweite Phase - dritte Phase	0,071	-0,113	0,254	0,067	16888,000	1,046	0,834	0,025
zweite Phase - vierte Phase	-0,064	-0,286	0,159	0,081	16888,000	-0,783	0,936	-0,023
zweite Phase - fünfte Phase	-0,007	-0,234	0,220	0,083	16888,000	-0,080	1,000	-0,002
dritte Phase - vierte Phase	-0,134	-0,371	0,103	0,087	16888,000	-1,547	0,532	-0,048
dritte Phase - fünfte Phase	-0,077	-0,318	0,164	0,088	16888,000	-0,875	0,906	-0,028
vierte Phase - fünfte Phase	0,057	-0,212	0,326	0,099	16888,000	0,579	0,978	0,021

	Tabelle 2d: COVID-19-bezogene Angst							
Vergleich	Schätzer / Differenz	Untere 95%-CI-Grenze	Obere 95%-CI-Grenze	Standardfehler	df	t-Ratio	p-Wert	d
erste Phase - zweite Phase	-0,694	-0,789	-0,599	0,035	16888,000	-19,898	0,000	-0,395
erste Phase - dritte Phase	-0,286	-0,395	-0,176	0,040	16888,000	-7,122	0,000	-0,163
erste Phase - vierte Phase	0,345	0,210	0,479	0,049	16888,000	6,995	0,000	0,196
erste Phase - fünfte Phase	0,690	0,553	0,828	0,050	16888,000	13,706	0,000	0,393
zweite Phase - dritte Phase	0,408	0,292	0,524	0,043	16888,000	9,584	0,000	0,232
zweite Phase - vierte Phase	1,039	0,898	1,179	0,051	16888,000	20,184	0,000	0,592
zweite Phase - fünfte Phase	1,384	1,241	1,528	0,053	16888,000	26,338	0,000	0,789
dritte Phase - vierte Phase	0,631	0,481	0,780	0,055	16888,000	11,496	0,000	0,359
dritte Phase - fünfte Phase	0,976	0,824	1,128	0,056	16888,000	17,512	0,000	0,556
vierte Phase - fünfte Phase	0,346	0,176	0,516	0,062	16888,000	5,548	0,000	0,197

	Tabelle 2e: Vertrauen in staatliche Maßnahmen in Bezug auf COVID-19							
Vergleich	Schätzer / Differenz	Untere 95%-CI-Grenze	Obere 95%-CI-Grenze	Standardfehler	df	t-Ratio	p-Wert	d
erste Phase - zweite Phase	-0,322	-0,397	-0,246	0,028	16888,000	-11,655	0,000	-0,231
erste Phase - dritte Phase	-0,598	-0,685	-0,512	0,032	16888,000	-18,839	0,000	-0,430
erste Phase - vierte Phase	-0,597	-0,704	-0,491	0,039	16888,000	-15,302	0,000	-0,430
erste Phase - fünfte Phase	-0,444	-0,553	-0,335	0,040	16888,000	-11,139	0,000	-0,320
zweite Phase - dritte Phase	-0,277	-0,368	-0,185	0,034	16888,000	-8,201	0,000	-0,199
zweite Phase - vierte Phase	-0,276	-0,387	-0,164	0,041	16888,000	-6,763	0,000	-0,198
zweite Phase - fünfte Phase	-0,123	-0,236	-0,009	0,042	16888,000	-2,945	0,027	-0,088
dritte Phase - vierte Phase	0,001	-0,117	0,119	0,043	16888,000	0,022	1,000	0,001
dritte Phase - fünfte Phase	0,154	0,034	0,274	0,044	16888,000	3,489	0,004	0,111
vierte Phase - fünfte Phase	0,153	0,018	0,288	0,049	16888,000	3,102	0,017	0,110

	Tabelle 2f: Subjektives Informiertheitslevel in Bezug auf COVID-19							
Vergleich	Schätzer / Differenz	Untere 95%-CI-Grenze	Obere 95%-CI-Grenze	Standardfehler	df	t-Ratio	p-Wert	d
erste Phase - zweite Phase	-0,371	-0,429	-0,314	0,021	16888,000	-17,710	0,000	-0,352
erste Phase - dritte Phase	-0,189	-0,255	-0,123	0,024	16888,000	-7,820	0,000	-0,179
erste Phase - vierte Phase	0,105	0,024	0,186	0,030	16888,000	3,528	0,004	0,099
erste Phase - fünfte Phase	0,191	0,109	0,274	0,030	16888,000	6,312	0,000	0,181
zweite Phase - dritte Phase	0,183	0,113	0,253	0,026	16888,000	7,135	0,000	0,173
zweite Phase - vierte Phase	0,476	0,392	0,561	0,031	16888,000	15,380	0,000	0,451
zweite Phase - fünfte Phase	0,563	0,477	0,649	0,032	16888,000	17,800	0,000	0,533
dritte Phase - vierte Phase	0,293	0,203	0,383	0,033	16888,000	8,891	0,000	0,278
dritte Phase - fünfte Phase	0,380	0,289	0,471	0,034	16888,000	11,332	0,000	0,360
vierte Phase - fünfte Phase	0,087	-0,016	0,189	0,037	16888,000	2,312	0,141	0,082

Tabelle 2g: Adhärentes Sicherheitsverhalten

Vergleich	Schätzer / Differenz	Untere 95%-CI-Grenze	Obere 95%-CI-Grenze	Standardfehler	df	t-Ratio	p-Wert	d
erste Phase - zweite Phase	-1,508	-1,584	-1,433	0,028	16888,000	-54,218	0,000	-1,077
erste Phase - dritte Phase	-1,374	-1,462	-1,287	0,032	16888,000	-42,943	0,000	-0,981
erste Phase - vierte Phase	-0,745	-0,853	-0,638	0,039	16888,000	-18,943	0,000	-0,532
erste Phase - fünfte Phase	-0,196	-0,305	-0,086	0,040	16888,000	-4,871	0,000	-0,140
zweite Phase - dritte Phase	0,134	0,041	0,227	0,034	16888,000	3,944	0,001	0,096
zweite Phase - vierte Phase	0,763	0,651	0,875	0,041	16888,000	18,588	0,000	0,545
zweite Phase - fünfte Phase	1,313	1,198	1,427	0,042	16888,000	31,301	0,000	0,937
dritte Phase - vierte Phase	0,629	0,510	0,749	0,044	16888,000	14,377	0,000	0,449
dritte Phase - fünfte Phase	1,179	1,057	1,300	0,044	16888,000	26,499	0,000	0,841
vierte Phase - fünfte Phase	0,549	0,414	0,685	0,050	16888,000	11,052	0,000	0,392

Tabelle 2h: %-Risiko, an COVID-19 zu erkranken

Vergleich	Schätzer / Differenz	Untere 95%-CI-Grenze	Obere 95%-CI-Grenze	Standardfehler	df	t-Ratio	p-Wert	d
erste Phase - zweite Phase	-7,671	-9,049	-6,293	0,505	16888,000	-15,187	0,000	-0,302
erste Phase - dritte Phase	-4,452	-6,037	-2,867	0,581	16888,000	-7,661	0,000	-0,175
erste Phase - vierte Phase	3,914	1,966	5,862	0,714	16888,000	5,480	0,000	0,154
erste Phase - fünfte Phase	13,098	11,107	15,089	0,730	16888,000	17,947	0,000	0,515
zweite Phase - dritte Phase	3,219	1,536	4,902	0,617	16888,000	5,218	0,000	0,127
zweite Phase - vierte Phase	11,585	9,551	13,619	0,745	16888,000	15,540	0,000	0,456
zweite Phase - fünfte Phase	20,769	18,692	22,846	0,761	16888,000	27,278	0,000	0,817
dritte Phase - vierte Phase	8,366	6,198	10,533	0,795	16888,000	10,529	0,000	0,329
dritte Phase - fünfte Phase	17,550	15,347	19,753	0,807	16888,000	21,734	0,000	0,690
vierte Phase - fünfte Phase	9,184	6,722	11,646	0,902	16888,000	10,176	0,000	0,361

Tabelle 2i: %-Risiko, im Falle einer Infektion einen schweren Verlauf zu erleben

Vergleich	Schätzer / Differenz	Untere 95%-CI-Grenze	Obere 95%-CI-Grenze	Standardfehler	df	t-Ratio	p-Wert	d
erste Phase - zweite Phase	-2,415	-3,613	-1,218	0,439	16888,000	-5,501	0,000	-0,109
erste Phase - dritte Phase	-2,517	-3,895	-1,139	0,505	16888,000	-4,982	0,000	-0,114
erste Phase - vierte Phase	0,067	-1,627	1,760	0,621	16888,000	0,107	1,000	0,003
erste Phase - fünfte Phase	0,859	-0,872	2,589	0,634	16888,000	1,353	0,658	0,039
zweite Phase - dritte Phase	-0,101	-1,564	1,362	0,536	16888,000	-0,189	1,000	-0,005
zweite Phase - vierte Phase	2,482	0,714	4,250	0,648	16888,000	3,830	0,001	0,112
zweite Phase - fünfte Phase	3,274	1,468	5,080	0,662	16888,000	4,947	0,000	0,148
dritte Phase - vierte Phase	2,583	0,699	4,468	0,691	16888,000	3,740	0,002	0,117
dritte Phase - fünfte Phase	3,375	1,460	5,290	0,702	16888,000	4,808	0,000	0,153
vierte Phase - fünfte Phase	0,792	-1,348	2,932	0,785	16888,000	1,009	0,851	0,036

Tabelle 2j: %-Risiko, im Falle einer Infektion zu sterben

Vergleich	Schätzer / Differenz	Untere 95%-CI-Grenze	Obere 95%-CI-Grenze	Standardfehler	df	t-Ratio	p-Wert	d
erste Phase - zweite Phase	-2,212	-3,201	-1,222	0,363	16888,000	-6,098	0,000	-0,121
erste Phase - dritte Phase	-2,886	-4,024	-1,748	0,417	16888,000	-6,918	0,000	-0,158
erste Phase - vierte Phase	-1,377	-2,776	0,022	0,513	16888,000	-2,685	0,056	-0,075
erste Phase - fünfte Phase	-2,213	-3,642	-0,783	0,524	16888,000	-4,223	0,000	-0,121
zweite Phase - dritte Phase	-0,674	-1,883	0,534	0,443	16888,000	-1,523	0,548	-0,037
zweite Phase - vierte Phase	0,835	-0,625	2,295	0,535	16888,000	1,560	0,523	0,046
zweite Phase - fünfte Phase	-0,001	-1,492	1,490	0,547	16888,000	-0,002	1,000	0,000
dritte Phase - vierte Phase	1,509	-0,047	3,065	0,570	16888,000	2,646	0,062	0,083
dritte Phase - fünfte Phase	0,673	-0,908	2,255	0,580	16888,000	1,161	0,773	0,037
vierte Phase - fünfte Phase	-0,836	-2,604	0,932	0,648	16888,000	-1,290	0,697	-0,046

Tabelle Z3: Regressionskoeffizienten. Links sind die jeweiligen Dummy-codierten unabhängigen Variablen verzeichnet. Die Referenzkategorie wurde mit angegeben. Jede Spalte bildet eine oben verzeichnete abhängige Variable ab. Zusätzlich zu den jeweiligen Regressionsgewichten ist das 95%-Konfidenzintervall angegeben.

Variable	Regressionsterm	Generalisierte Angst (GAD-7)	Depressive Symptome (PHQ-2)	COVID-19-bezogene Angst	Vertrauen in politische Maßnahmen	Subjektives Informiertheitslevel	Adhärentes Sicherheitsver-halten	%-Risiko, an COVID-19 zu erkranken	%-Risiko für schweren Verlauf	%-Risiko, zu sterben
	Interzept	5.69 ***<br>[5.40, 5.98]	1.34 ***<br>[1.25, 1.43]	4.20 ***<br>[4.08, 4.31]	4.67 ***<br>[4.58, 4.76]	5.81 ***<br>[5.74, 5.88]	5.04 ***<br>[4.95, 5.13]	48.85 ***<br>[47.18, 50.52]	15.98 ***<br>[14.53, 17.43]	4.08 ***<br>[2.88, 5.28]
Zeit (ref: Phase 1)	Phase 2	1.04 ***<br>[0.86, 1.22]	0.52 ***<br>[0.46, 0.57]	-0.77 ***<br>[-0.84, -0.69]	0.37 ***<br>[0.31, 0.42]	-0.27 ***<br>[-0.31, -0.23]	-0.12 ***<br>[-0.17, -0.06]	-11.95 ***<br>[-12.97, -10.92]	-1.33 **<br>[-2.22, -0.44]	1.14 **<br>[0.40, 1.87]
	Phase 3	-1.03 ***<br>[-1.20, -0.85]	-0.20 ***<br>[-0.25, -0.14]	-0.62 ***<br>[-0.68, -0.55]	-0.33 ***<br>[-0.38, -0.27]	-0.27 ***<br>[-0.32, -0.23]	-1.23 ***<br>[-1.29, -1.18]	-10.38 ***<br>[-11.37, -9.40]	-2.43 ***<br>[-3.29, -1.57]	-1.32 ***<br>[-2.03, -0.61]
	Phase 4	0.23 *<br>[0.05, 0.41]	-0.07 *<br>[-0.13, -0.02]	0.44 ***<br>[0.37, 0.51]	-0.03<br>[-0.09, 0.02]	0.24 ***<br>[0.20, 0.28]	0.54 ***<br>[0.49, 0.60]	3.19 ***<br>[2.16, 4.21]	1.30 **<br>[0.41, 2.19]	1.23 **<br>[0.49, 1.96]
	Phase 5	-0.21 *<br>[-0.38, -0.04]	0.00<br>[-0.05, 0.06]	-0.04<br>[-0.11, 0.02]	0.04<br>[-0.01, 0.10]	-0.02<br>[-0.06, 0.03]	-0.07 *<br>[-0.12, -0.01]	-0.17<br>[-1.15, 0.81]	0.58<br>[-0.27, 1.43]	0.62<br>[-0.09, 1.32]
Geschlecht<br>(ref: Weiblich)	Männlich	-1.14 ***<br>[-1.29, -0.99]	-0.07 **<br>[-0.12, -0.02]	-0.64 ***<br>[-0.70, -0.58]	-0.14 ***<br>[-0.18, -0.09]	-0.15 ***<br>[-0.19, -0.12]	-0.46 ***<br>[-0.51, -0.41]	-2.11 ***<br>[-2.98, -1.25]	-5.12 ***<br>[-5.88, -4.37]	-3.16 ***<br>[-3.78, -2.54]
	Divers	-0.23<br>[-1.39, 0.94]	0.45 *<br>[0.09, 0.82]	-0.59 *<br>[-1.05, -0.13]	-0.59 **<br>[-0.96, -0.23]	-0.38 **<br>[-0.65, -0.10]	-0.79 ***<br>[-1.15, -0.42]	-5.42<br>[-12.05, 1.21]	-6.05 *<br>[-11.81, -0.28]	-4.13<br>[-8.89, 0.63]
Alter<br>(ref: 18-24 Jahre)	25-34 Jahre	-0.33 *<br>[-0.59, -0.06]	-0.25 ***<br>[-0.34, -0.17]	0.08<br>[-0.02, 0.19]	-0.08<br>[-0.16, 0.00]	-0.00<br>[-0.07, 0.06]	0.07<br>[-0.02, 0.15]	0.21<br>[-1.30, 1.73]	1.81 **<br>[0.50, 3.13]	1.99 ***<br>[0.90, 3.08]
	35-44 Jahre	-0.28<br>[-0.57, 0.01]	-0.29 ***<br>[-0.38, -0.20]	0.39 ***<br>[0.28, 0.50]	-0.01<br>[-0.10, 0.08]	0.03<br>[-0.04, 0.09]	0.36 ***<br>[0.27, 0.45]	0.46<br>[-1.18, 2.09]	4.76 ***<br>[3.33, 6.18]	3.50 ***<br>[2.33, 4.68]
	45-54 Jahre	-0.74 ***<br>[-1.03, -0.44]	-0.38 ***<br>[-0.47, -0.29]	0.39 ***<br>[0.27, 0.51]	0.15 **<br>[0.05, 0.24]	0.12 ***<br>[0.05, 0.19]	0.41 ***<br>[0.32, 0.51]	-1.57<br>[-3.26, 0.12]	6.34 ***<br>[4.87, 7.80]	5.15 ***<br>[3.94, 6.36]
	55-64 Jahre	-1.39 ***<br>[-1.71, -1.08]	-0.46 ***<br>[-0.56, -0.36]	0.48 ***<br>[0.35, 0.60]	0.35 ***<br>[0.25, 0.45]	0.18 ***<br>[0.11, 0.26]	0.53 ***<br>[0.43, 0.63]	-6.20 ***<br>[-7.99, -4.41]	10.00 ***<br>[8.44, 11.55]	7.29 ***<br>[6.00, 8.57]
	65-74 Jahre	-2.28 ***<br>[-2.72, -1.85]	-0.70 ***<br>[-0.84, -0.57]	0.50 ***<br>[0.33, 0.67]	0.69 ***<br>[0.56, 0.83]	0.31 ***<br>[0.21, 0.41]	0.58 ***<br>[0.45, 0.72]	-10.91 ***<br>[-13.38, -8.43]	12.79 ***<br>[10.64, 14.94]	9.95 ***<br>[8.18, 11.73]
	Alter 75+	-2.23 ***<br>[-3.03, -1.43]	-0.76 ***<br>[-1.01, -0.51]	0.78 ***<br>[0.47, 1.09]	0.99 ***<br>[0.74, 1.24]	0.25 *<br>[0.06, 0.44]	0.80 ***<br>[0.55, 1.05]	-13.53 ***<br>[-18.08, -8.99]	25.38 ***<br>[21.43, 29.33]	24.19 ***<br>[20.93, 27.46]
Bildung<br>(ref: Universitäts-abschluss)	Abitur	0.13<br>[-0.04, 0.31]	0.09 **<br>[0.04, 0.14]	-0.15 ***<br>[-0.22, -0.08]	-0.24 ***<br>[-0.30, -0.19]	-0.13 ***<br>[-0.17, -0.09]	-0.13 ***<br>[-0.18, -0.08]	-1.71 ***<br>[-2.69, -0.73]	1.42 **<br>[0.56, 2.27]	1.71 ***<br>[1.00, 2.41]
	Realschule	0.32 **<br>[0.12, 0.51]	0.14 ***<br>[0.08, 0.21]	-0.19 ***<br>[-0.27, -0.11]	-0.46 ***<br>[-0.52, -0.40]	-0.29 ***<br>[-0.34, -0.25]	-0.21 ***<br>[-0.27, -0.15]	-3.34 ***<br>[-4.47, -2.21]	5.16 ***<br>[4.18, 6.15]	4.80 ***<br>[3.99, 5.61]
	Hauptschule	0.39 *<br>[0.04, 0.73]	0.19 ***<br>[0.08, 0.30]	-0.04<br>[-0.17, 0.10]	-0.46 ***<br>[-0.56, -0.35]	-0.45 ***<br>[-0.53, -0.37]	-0.16 **<br>[-0.26, -0.05]	-5.62 ***<br>[-7.58, -3.66]	8.35 ***<br>[6.64, 10.05]	9.81 ***<br>[8.41, 11.22]
	Kein Abschluss	0.47<br>[-0.72, 1.65]	0.43 *<br>[0.06, 0.80]	-0.12<br>[-0.58, 0.35]	-0.33<br>[-0.70, 0.04]	-0.84 ***<br>[-1.12, -0.56]	-0.36<br>[-0.73, 0.01]	-8.06 *<br>[-14.79, -1.33]	12.59 ***<br>[6.74, 18.44]	20.06 ***<br>[15.23, 24.89]
	Sonstige	0.52<br>[-0.01, 1.06]	0.11<br>[-0.06, 0.27]	-0.03<br>[-0.24, 0.18]	-0.29 ***<br>[-0.46, -0.12]	-0.10<br>[-0.23, 0.03]	-0.08<br>[-0.24, 0.09]	-3.29 *<br>[-6.34, -0.25]	3.85 **<br>[1.20, 6.49]	3.68 ***<br>[1.50, 5.87]
Beruf<br>(ref: Andere)	Arbeitslos	0.43 ***<br>[0.18, 0.68]	0.21 ***<br>[0.13, 0.29]	0.06<br>[-0.04, 0.16]	-0.07<br>[-0.14, 0.01]	0.03<br>[-0.03, 0.09]	0.12 **<br>[0.04, 0.19]	-1.98 **<br>[-3.39, -0.57]	4.02 ***<br>[2.79, 5.25]	3.71 ***<br>[2.69, 4.72]
	Arzt	-0.52 **<br>[-0.90, -0.14]	-0.17 **<br>[-0.29, -0.05]	-0.30 ***<br>[-0.45, -0.15]	-0.16 **<br>[-0.28, -0.05]	-0.03<br>[-0.12, 0.06]	-0.17 **<br>[-0.29, -0.05]	6.26 ***<br>[4.11, 8.42]	-7.72 ***<br>[-9.59, -5.84]	-2.49 **<br>[-4.04, -0.95]
	Pflege	-0.51 ***<br>[-0.75, -0.28]	-0.18 ***<br>[-0.25, -0.10]	-0.27 ***<br>[-0.36, -0.17]	-0.28 ***<br>[-0.35, -0.20]	-0.01<br>[-0.06, 0.05]	-0.16 ***<br>[-0.23, -0.08]	3.71 ***<br>[2.38, 5.03]	-2.80 ***<br>[-3.95, -1.64]	-1.30 **<br>[-2.25, -0.35]
	Rettungsdienst / Feuerwehr / Polizei	-1.01 ***<br>[-1.48, -0.54]	-0.25 **<br>[-0.39, -0.10]	-0.23 *<br>[-0.41, -0.04]	-0.39 ***<br>[-0.54, -0.24]	0.04<br>[-0.07, 0.15]	-0.25 ***<br>[-0.40, -0.11]	4.87 ***<br>[2.18, 7.56]	-1.94<br>[-4.28, 0.40]	-0.77<br>[-2.70, 1.16]
	Schüler / Student	0.28 *<br>[0.01, 0.55]	0.09 *<br>[0.01, 0.18]	0.09<br>[-0.02, 0.20]	0.20 ***<br>[0.12, 0.28]	0.10 **<br>[0.04, 0.16]	0.07<br>[-0.01, 0.16]	-1.64 *<br>[-3.17, -0.10]	-1.07<br>[-2.40, 0.27]	-0.00<br>[-1.10, 1.10]
Psychische Erkrankung<br>(ref: nein)	Ja	4.48 ***<br>[4.27, 4.69]	1.53 ***<br>[1.46, 1.60]	0.36 ***<br>[0.28, 0.44]	-0.21 ***<br>[-0.28, -0.15]	0.01<br>[-0.04, 0.06]	0.14 ***<br>[0.07, 0.20]	2.22 ***<br>[1.01, 3.43]	3.39 ***<br>[2.34, 4.44]	3.03 ***<br>[2.17, 3.90]
Gemeindegröße (ref: Großstadt)	Mittelgroße Stadt	0.11<br>[-0.06, 0.28]	0.03<br>[-0.02, 0.09]	-0.06<br>[-0.13, 0.00]	-0.13 ***<br>[-0.19, -0.08]	-0.12 ***<br>[-0.16, -0.08]	0.02<br>[-0.03, 0.07]	-0.27<br>[-1.24, 0.69]	0.63<br>[-0.22, 1.47]	0.40<br>[-0.29, 1.09]
	Kleinstadt	0.16<br>[-0.06, 0.38]	0.04<br>[-0.03, 0.11]	-0.07<br>[-0.16, 0.02]	-0.09 **<br>[-0.16, -0.02]	-0.09 ***<br>[-0.14, -0.04]	0.11 **<br>[0.04, 0.18]	-0.11<br>[-1.37, 1.16]	0.08<br>[-1.02, 1.18]	0.12<br>[-0.79, 1.03]
	Landkreis	0.20<br>[-0.03, 0.42]	-0.01<br>[-0.08, 0.06]	-0.14 **<br>[-0.23, -0.06]	-0.24 ***<br>[-0.31, -0.17]	-0.12 ***<br>[-0.17, -0.06]	0.06<br>[-0.01, 0.13]	-0.38<br>[-1.65, 0.90]	0.35<br>[-0.76, 1.45]	0.29<br>[-0.62, 1.20]
Körperliche Erkrankung<br>(ref: nein)	Ja	0.49 ***<br>[0.31, 0.66]	0.10 ***<br>[0.05, 0.16]	0.30 ***<br>[0.23, 0.37]	-0.12 ***<br>[-0.18, -0.07]	0.03<br>[-0.01, 0.07]	0.16 ***<br>[0.11, 0.22]	2.73 ***<br>[1.74, 3.72]	14.03 ***<br>[13.17, 14.89]	8.67 ***<br>[7.96, 9.38]
	N	16916	16916	16916	16916	16916	16916	16916	16916	16916
	R2	0.18	0.18	0.11	0.08	0.06	0.23	0.08	0.17	0.14

*** p < 0.001; ** p < 0.01; * p < 0.05.

Tab. Z4: Robuste Regressionen

Tabelle Z4a: Regressionstabelle / Robuste Regression

Variable	Regressionsterm	Generalisierte Angst (GAD-7)	Depressive Symptome (PHQ-2)
	Interzept	5.69 ***	1.34 ***
		[5.38. 6.00]	[1.24. 1.44]
Zeit (ref: Phase 1)	Phase 2	1.04 ***	0.52 ***
		[0.85. 1.22]	[0.45. 0.58]
	Phase 3	-1.03 ***	-0.20 ***
		[-1.21. -0.84]	[-0.26. -0.14]
	Phase 4	0.23 *	-0.07 *
		[0.03. 0.42]	[-0.14. -0.01]
	Phase 5	-0.21 *	0.00
		[-0.40. -0.02]	[-0.06. 0.06]
Geschlecht (ref: Weiblich)	Männlich	-1.14 ***	-0.07 **
		[-1.29. -0.99]	[-0.12. -0.03]
	Divers	-0.23	0.45 *
		[-1.55. 1.10]	[0.00. 0.91]
Alter (ref: 18-24 Jahre)	25-34 Jahre	-0.33 *	-0.25 ***
		[-0.61. -0.05]	[-0.35. -0.16]
	35-44 Jahre	-0.28	-0.29 ***
		[-0.58. 0.02]	[-0.39. -0.19]
	45-54 Jahre	-0.74 ***	-0.38 ***
		[-1.05. -0.43]	[-0.48. -0.28]
	55-64 Jahre	-1.39 ***	-0.46 ***
		[-1.72. -1.07]	[-0.57. -0.36]
	65-74 Jahre	-2.28 ***	-0.70 ***
		[-2.70. -1.86]	[-0.84. -0.57]
	Alter 75+	-2.23 ***	-0.76 ***
		[-2.91. -1.54]	[-0.99. -0.52]
Bildung (ref: Universitätsabschluss)	Abitur	0.13	0.09 **
		[-0.04. 0.31]	[0.04. 0.14]
	Realschule	0.32 **	0.14 ***
		[0.11. 0.52]	[0.08. 0.21]
	Hauptschule	0.39 *	0.19 **
		[0.04. 0.74]	[0.07. 0.30]
	Kein Abschluss	0.47	0.43
		[-0.94. 1.88]	[-0.07. 0.92]
	Sonstige	0.52	0.11
		[-0.09. 1.14]	[-0.08. 0.29]
Beruf (ref: Andere)	Arbeitslos	0.43 **	0.21 ***
		[0.16. 0.70]	[0.12. 0.30]
	Arzt	-0.52 **	-0.17 ***
		[-0.84. -0.20]	[-0.26. -0.07]
	Pflege	-0.51 ***	-0.18 ***
		[-0.74. -0.29]	[-0.24. -0.11]
	Rettungsdienst / Feuerwehr / Polizei	-1.01 ***	-0.25 ***
		[-1.44. -0.58]	[-0.37. -0.12]
	Schüler / Student	0.28	0.09 *
		[-0.00. 0.56]	[0.00. 0.18]
Psychischer Erkrankung (ref: nein)	Ja	4.48 ***	1.53 ***
		[4.22. 4.73]	[1.44. 1.62]
Gemeindegröße (ref: Großstadt)	Mittelgroße Stadt	0.11	0.03
		[-0.06. 0.28]	[-0.02. 0.09]
	Kleinstadt	0.16	0.04
		[-0.06. 0.39]	[-0.03. 0.11]
	Landkreis	0.20	-0.01
		[-0.03. 0.43]	[-0.09. 0.06]
Körperliche Erkrankung (ref: nein):	Ja	0.49 ***	0.10 ***
		[0.31. 0.67]	[0.05. 0.16]
	N	16916	16916
	R2	0.18	0.18

Die Standardfehler sind robust gegen Heteroskedastizität. *** p < 0.001; ** p < 0.01; * p < 0.05.

4b: Regressionstabelle / Robuste Regression

Variable	Regressionsterm	COVID-19-bezogene Angst	Vertrauen in politische Maßnahmen	Subjektives Informiertheitslevel	Adhärentes Sicherheitsverhalten
	Interzept	4.20 ***	4.67 ***	5.81 ***	5.04 ***
		[4.08, 4.31]	[4.58, 4.76]	[5.74, 5.88]	[4.95, 5.13]
Zeit (ref: Phase 1)	Phase 2	-0.77 ***	0.37 ***	-0.27 ***	-0.12 ***
		[-0.84, -0.69]	[0.31, 0.43]	[-0.32, -0.22]	[-0.18, -0.05]
	Phase 3	-0.62 ***	-0.33 ***	-0.27 ***	-1.23 ***
		[-0.69, -0.54]	[-0.38, -0.27]	[-0.32, -0.23]	[-1.29, -1.17]
	Phase 4	0.44 ***	-0.03	0.24 ***	0.54 ***
		[0.36, 0.51]	[-0.09, 0.02]	[0.19, 0.29]	[0.48, 0.61]
	Phase 5	-0.04	0.04	-0.02	-0.07 *
		[-0.12, 0.03]	[-0.01, 0.10]	[-0.06, 0.03]	[-0.12, -0.01]
Geschlecht (ref: Weiblich)	Männlich	-0.64 ***	-0.14 ***	-0.15 ***	-0.46 ***
		[-0.70, -0.58]	[-0.18, -0.09]	[-0.19, -0.12]	[-0.51, -0.41]
	Divers	-0.59 *	-0.59 **	-0.38 *	-0.79 ***
		[-1.04, -0.13]	[-1.03, -0.16]	[-0.70, -0.05]	[-1.18, -0.39]
Alter (ref: 18-24 Jahre)	25-34 Jahre	0.08	-0.08 *	-0.00	0.07
		[-0.02, 0.19]	[-0.16, -0.00]	[-0.06, 0.06]	[-0.02, 0.15]
	35-44 Jahre	0.39 ***	-0.01	0.03	0.36 ***
		[0.28, 0.50]	[-0.10, 0.08]	[-0.04, 0.09]	[0.27, 0.45]
	45-54 Jahre	0.39 ***	0.15 **	0.12 ***	0.41 ***
		[0.27, 0.51]	[0.06, 0.24]	[0.05, 0.19]	[0.32, 0.51]
	55-64 Jahre	0.48 ***	0.35 ***	0.18 ***	0.53 ***
		[0.35, 0.60]	[0.25, 0.45]	[0.11, 0.26]	[0.43, 0.63]
	65-74 Jahre	0.50 ***	0.69 ***	0.31 ***	0.58 ***
		[0.33, 0.67]	[0.56, 0.82]	[0.21, 0.41]	[0.45, 0.72]
	Alter 75+	0.78 ***	0.99 ***	0.25 *	0.80 ***
		[0.45, 1.10]	[0.74, 1.25]	[0.05, 0.45]	[0.54, 1.06]
Bildung (ref: Universitätsabschluss)	Abitur	-0.15 ***	-0.24 ***	-0.13 ***	-0.13 ***
		[-0.22, -0.08]	[-0.30, -0.19]	[-0.17, -0.09]	[-0.18, -0.08]
	Realschule	-0.19 ***	-0.46 ***	-0.29 ***	-0.21 ***
		[-0.27, -0.11]	[-0.52, -0.39]	[-0.34, -0.24]	[-0.27, -0.14]
	Hauptschule	-0.04	-0.46 ***	-0.45 ***	-0.16 **
		[-0.18, 0.11]	[-0.57, -0.34]	[-0.54, -0.35]	[-0.27, -0.04]
	Kein Abschluss	-0.12	-0.33	-0.84 ***	-0.36
		[-0.65, 0.42]	[-0.81, 0.15]	[-1.30, -0.38]	[-0.81, 0.09]
	Sonstige	-0.03	-0.29 ***	-0.10	-0.08
		[-0.24, 0.19]	[-0.46, -0.12]	[-0.23, 0.03]	[-0.24, 0.08]
Beruf (ref: Andere)	Arbeitslos	0.06	-0.07	0.03	0.12 **
		[-0.04, 0.16]	[-0.15, 0.01]	[-0.03, 0.09]	[0.04, 0.19]
	Arzt	-0.30 ***	-0.16 **	-0.03	-0.17 **
		[-0.45, -0.15]	[-0.28, -0.04]	[-0.12, 0.07]	[-0.29, -0.05]
	Pflege	-0.27 ***	-0.28 ***	-0.01	-0.16 ***
		[-0.36, -0.17]	[-0.35, -0.20]	[-0.07, 0.05]	[-0.23, -0.08]
	Rettungsdienst / Feuerwehr / Polizei	-0.23 *	-0.39 ***	0.04	-0.25 **
		[-0.42, -0.03]	[-0.55, -0.23]	[-0.08, 0.17]	[-0.42, -0.09]
	Schüler / Student	0.09	0.20 ***	0.10 **	0.07
		[-0.01, 0.20]	[0.12, 0.28]	[0.04, 0.16]	[-0.01, 0.16]
Psychischer Erkrankung (ref: nein)	Ja	0.36 ***	-0.21 ***	0.01	0.14 ***
		[0.28, 0.44]	[-0.28, -0.15]	[-0.04, 0.06]	[0.07, 0.20]
Gemeindegröße (ref: Großstadt)	Mittelgroße Stadt	-0.06	-0.13 ***	-0.12 ***	0.02
		[-0.13, 0.00]	[-0.19, -0.08]	[-0.16, -0.08]	[-0.03, 0.08]
	Kleinstadt	-0.07	-0.09 **	-0.09 **	0.11 **
		[-0.16, 0.02]	[-0.16, -0.02]	[-0.14, -0.03]	[0.04, 0.18]
	Landkreis	-0.14 **	-0.24 ***	-0.12 ***	0.06
		[-0.24, -0.05]	[-0.31, -0.17]	[-0.17, -0.06]	[-0.02, 0.13]
Körperliche Erkrankung (ref nein)	Ja	0.30 ***	-0.12 ***	0.03	0.16 ***
		[0.23, 0.37]	[-0.18, -0.07]	[-0.02, 0.07]	[0.11, 0.22]
	N	16916	16916	16916	16916
	R2	0.11	0.08	0.06	0.23

Die Standardfehler sind robust gegen Heteroskedastizität.*** $p < 0.001$; ** $p < 0.01$; * $p < 0.05$.

4c: Regressionstabelle / Robuste Regression

Variable	Regressionsterm	%-Risiko, an COVID-19 zu erkranken	%-Risiko für schweren Verlauf	%-Risiko, zu sterben
	Interzept	48.85 ***	15.98 ***	4.08 ***
		[47.16, 50.53]	[14.66, 17.30]	[3.06, 5.10]
Zeit (ref: Phase 1)	Phase 2	-11.95 ***	-1.33 **	1.14 **
		[-13.04, -10.86]	[-2.28, -0.38]	[0.34, 1.93]
	Phase 3	-10.38 ***	-2.43 ***	-1.32 ***
		[-11.44, -9.33]	[-3.34, -1.52]	[-2.08, -0.56]
	Phase 4	3.19 ***	1.30 **	1.23 **
		[2.10, 4.27]	[0.39, 2.21]	[0.47, 1.99]
	Phase 5	-0.17	0.58	0.62
		[-1.20, 0.86]	[-0.30, 1.45]	[-0.11, 1.35]
Geschlecht (ref: Weiblich)	Männlich	-2.11 ***	-5.12 ***	-3.16 ***
		[-3.01, -1.22]	[-5.87, -4.38]	[-3.77, -2.55]
	Divers	-5.42	-6.05 *	-4.13
		[-13.40, 2.57]	[-11.90, -0.20]	[-9.21, 0.94]
Alter (ref: 18-24 Jahre)	25-34 Jahre	0.21	1.81 **	1.99 ***
		[-1.32, 1.75]	[0.64, 2.99]	[1.10, 2.88]
	35-44 Jahre	0.46	4.76 ***	3.50 ***
		[-1.21, 2.12]	[3.45, 6.06]	[2.49, 4.52]
	45-54 Jahre	-1.57	6.34 ***	5.15 ***
		[-3.29, 0.15]	[4.96, 7.71]	[4.05, 6.25]
	55-64 Jahre	-6.20 ***	10.00 ***	7.29 ***
		[-8.00, -4.40]	[8.47, 11.53]	[6.03, 8.54]
	65-74 Jahre	-10.91 ***	12.79 ***	9.95 ***
		[-13.30, -8.51]	[10.43, 15.14]	[7.86, 12.04]
	Alter 75+	-13.53 ***	25.38 ***	24.19 ***
		[-17.98, -9.09]	[20.31, 30.46]	[18.63, 29.75]
Bildung- (ref: Universitätsabschluss)	Abitur	-1.71 ***	1.42 ***	1.71 ***
		[-2.69, -0.74]	[0.59, 2.24]	[1.05, 2.37]
	Realschule	-3.34 ***	5.16 ***	4.80 ***
		[-4.49, -2.20]	[4.10, 6.22]	[3.90, 5.69]
	Hauptschule	-5.62 ***	8.35 ***	9.81 ***
		[-7.61, -3.62]	[6.25, 10.44]	[7.82, 11.81]
	Kein Abschluss	-8.06	12.59 **	20.06 ***
		[-16.49, 0.38]	[4.77, 20.41]	[11.52, 28.60]
	Sonstige	-3.29 *	3.85 **	3.68 **
		[-6.50, -0.08]	[0.92, 6.77]	[1.10, 6.27]
Beruf (ref: Andere)	Arbeitslos	-1.98 **	4.02 ***	3.71 ***
		[-3.38, -0.58]	[2.55, 5.49]	[2.37, 5.05]
	Arzt	6.26 ***	-7.72 ***	-2.49 ***
		[3.97, 8.55]	[-9.24, -6.20]	[-3.78, -1.21]
	Pflege	3.71 ***	-2.80 ***	-1.30 **
		[2.35, 5.06]	[-3.95, -1.64]	[-2.28, -0.33]
	Rettungsdienst / Feuerwehr / Polizei	4.87 ***	-1.94	-0.77
		[2.11, 7.63]	[-4.00, 0.11]	[-2.48, 0.95]
	Schüler / Student	-1.64 *	-1.07	-0.00
		[-3.17, -0.10]	[-2.26, 0.13]	[-0.93, 0.92]
Psychischer Erkrankung (ref: nein)	Ja	2.22 ***	3.39 ***	3.03 ***
		[1.05, 3.39]	[2.26, 4.52]	[2.03, 4.03]
Gemeindegröße (ref: Großstadt)	Mittelgroße Stadt	-0.27	0.63	0.40
		[-1.24, 0.69]	[-0.22, 1.47]	[-0.30, 1.10]
	Kleinstadt	-0.11	0.08	0.12
		[-1.38, 1.16]	[-1.05, 1.21]	[-0.83, 1.07]
	Landkreis	-0.38	0.35	0.29
		[-1.70, 0.95]	[-0.82, 1.52]	[-0.69, 1.27]
Körperliche Erkrankung (ref: nein)	Ja	2.73 ***	14.03 ***	8.67 ***
		[1.75, 3.70]	[13.00, 15.06]	[7.77, 9.57]
	N	16916	16916	16916
	R2	0.08	0.17	0.14

Die Standardfehler sind robust gegen Heteroskedastizität.*** p < 0.001; ** p < 0.01; * p < 0.05.
